# Personal characteristics and emotions accompanying nurses and midwives in caring for a newborn with a lethal defect

**DOI:** 10.1038/s41598-025-16322-9

**Published:** 2025-08-29

**Authors:** Katarzyna Urbańska, Agnieszka Drosdzol-Cop, Beata Naworska

**Affiliations:** 1 Neonatology Unit, BCM The Guardian Angels Hospital of the Brothers Hospitallers of St. John of God in Katowice, Markiefki 87, Katowice, 40-211 Poland; 2 Chair and Department of Gynecology, Obstetrics and Oncological Gynecology, Medical University of Silesia in Katowice, Katowice, 40-751 Poland; 3 Department of Gynaecology and Obstetrics, Faculty of Health Sciences in Katowice, Medical University of Silesia in Katowice, Katowice, 40-751 Poland

**Keywords:** Emotions, Personal characteristics, Nurses, Midwifes, Lethal defects, Psychology, Medical research

## Abstract

The aim of this study was to assess how professional experience, workplace setting, and personality traits influence the type and intensity of emotions experienced by nurses and midwives while caring for newborns with lethal congenital anomalies. A quantitative, cross-sectional study was conducted between April and June 2023 in five level II and III referral hospitals located in the Silesian Voivodeship, southern Poland. A total of 307 nurses and midwives meeting the inclusion criteria participated in the study. Data were collected using an original questionnaire comprising sociodemographic variables, self-assessed personality traits, and a scale measuring the type and intensity of emotions. Statistical analysis was conducted using Pearson’s and Spearman’s correlation coefficients (α = 0.05). The results indicated that longer professional experience was associated with increased empathy, patience, and sadness, and with decreased anger and emotional detachment. Higher levels of self-reported discomfort and frequent exposure to neonatal death were significantly correlated with intensified experiences of despair, fear, and helplessness. The findings highlight the complexity of emotional responses among perinatal healthcare professionals and underline the need for systemic emotional support and mental health strategies aimed at preventing burnout in emotionally demanding clinical settings.

## Introduction

The nursing and midwifery professions are not only associated with the responsibility of providing patient care but also with the necessity of coping with high levels of occupational stress. This stress stems from both physical and emotional aspects of the work, including demanding bedside care, direct interactions with patients’ families, tensions within therapeutic teams, and constant exposure to human suffering and death^1^. These roles require not only advanced professional competence but also psychological resilience, empathy, and the ability to manage emotions effectively in critical situations.

One of the most emotionally challenging areas of clinical care involves supporting terminally ill patients, including neonates diagnosed with lethal congenital anomalies. Contact with a dying patient—especially in the context of perinatal palliative care—becomes a source of profound emotional experience, which may lead to serious psychological consequences such as sadness, helplessness, guilt, or depressive symptoms^2–5^. Nurses and midwives who accompany patients and their families during the final stages of life are not merely observers of suffering but are emotionally involved participants in this process.

Perinatal palliative care, in particular—which encompasses Neonatal Intensive Care Units (NICUs), maternity wards, delivery rooms, and perinatal hospices—demands constant balancing between professionalism and deeply human experiences of grief and loss among families losing their newborns^6–10^. As nursing is a predominantly female profession, it is also burdened with social and cultural expectations of emotional engagement and empathy, which may exacerbate the effects of chronic occupational stress^11,12^.

Lethal congenital anomalies are severe developmental disorders with an extremely poor prognosis, typically leading to fetal loss, stillbirth, or death shortly after birth, regardless of the medical interventions provided^13^. These include, among others, trisomies of chromosomes 13 and 18, severe skeletal dysplasias, major brain malformations, renal agenesis, and cardiac anomalies such as hypoplastic lungs or absence of the heart. In the absence of effective causal treatment, the scope of medical intervention is limited to alleviating suffering and supporting families through the grieving process.

In caring for neonates with lethal anomalies, the individual characteristics of nurses and midwives—such as empathy, psychological resilience, and stress coping abilities—play a crucial role. A high level of empathy, while enhancing the quality of care, may paradoxically increase the risk of emotional exhaustion and burnout if not accompanied by adequate psychological support^14^. Conversely, psychological resilience serves as a protective buffer, enabling more effective emotional regulation in the face of chronic stress^14^.

Burnout is one of the most serious consequences of chronic workplace stress. It is characterised by emotional exhaustion, depersonalisation, and a diminished sense of personal accomplishment^15^. Within the context of caring for dying neonates, burnout may lead to a significant decline in care quality, loss of empathy, and even withdrawal from the profession. Personality traits such as perfectionism, a strong need to help others, or a tendency to assume responsibility may further exacerbate the risk of burnout.

Another phenomenon affecting the mental wellbeing of healthcare staff is moral distress—the psychological conflict that arises when one is forced to make decisions that contradict their personal values, beliefs, or ethical standards. In the care of neonates with lethal conditions, nurses often face moral dilemmas regarding the withdrawal of life-sustaining treatment or the need to emotionally support families through the dying process^16^. These situations may provoke intense internal conflict and long-term psychological strain.

Despite a growing body of literature exploring the emotional and psychological dimensions of nursing and midwifery in palliative care, comprehensive analyses that integrate professional experience, work environment, and personality traits remain scarce. Most previous studies have focused on isolated variables, limiting the development of effective preventive and interventional strategies.

This study seeks to fill that gap by examining the emotional experiences of nurses and midwives caring for neonates with lethal congenital anomalies. The objective is to identify both professional and individual factors that influence these emotional responses, including the frequency of contact with terminally ill patients and the level of exposure to neonatal death. Understanding the interplay of these variables may serve as a foundation for developing effective psychological support strategies and improving the quality of perinatal palliative care in clinical practice.

## Research aim

The primary aim of this study was to analyse the impact of professional experience (both overall and unit-specific), workplace setting, and personality traits on the type and intensity of emotions experienced by nurses and midwives while providing care for neonates with lethal congenital anomalies. Additionally, the study sought to determine how the frequency of contact with terminal patients, exposure to neonatal death, and subjective levels of discomfort affect the emotional condition of healthcare personnel. The research also aimed to identify occupational and individual factors that may exacerbate or alleviate emotional burden in the context of perinatal palliative care.

## Research methodology

### Methodology

The study was conducted in the second quarter of 2023 in the Silesian Voivodeship in southern Poland. It had a quantitative, cross-sectional design and was carried out using a diagnostic survey method with the application of an original questionnaire.

### Contextual background

The research context was closely related to the three-tier perinatal care system in force in Poland, where level II and III facilities play a key role in the care of newborns with lethal anomalies^17,18^. This system differentiates medical facilities based on their level of reference, defining the scope of care they provide:

- **Level I** – provides basic care for women with physiological pregnancies and for healthy newborns,
- **Level II** – offers care for medium-risk pregnancies and for newborns with mild health issues,
- **Level III** – delivers highly specialised care for high-risk pregnancies and newborns requiring intensive care, including those with lethal anomalies.

There are currently 59 hospitals with level III reference status in Poland, seven of which are located in the Silesian Voivodeship. All level III facilities in the region were invited to participate in the study; four agreed to take part. In order to maintain an appropriate sample size and diversity, one level II hospital was also included. This facility operates a perinatal hospice within its structure, enabling it to provide care for newborns diagnosed with lethal anomalies.

### Organisation and data collection

Data collection was carried out between 1 April and 30 June 2023. Paper versions of the questionnaire were distributed, each accompanied by instructions and an envelope to ensure anonymity. In each participating facility, locked boxes were placed for respondents to return completed questionnaires in sealed envelopes. Only the research team had access to the keys. Questionnaires were collected twice a week.

A total of 340 questionnaires were distributed. Of these, 316 were returned, and 307 were deemed complete and included in the final analysis.

### Sampling procedure

A purposive sampling method was applied. Participants were eligible for inclusion in the study if they met the following criteria:

- nursing or midwifery qualification,
- valid professional licence,
- employment in one of the following departments: Neonatology, Neonatal Intensive Care Unit, Neonatal Pathology, Obstetrics, Labour Ward, or Perinatal Hospice,

Individuals not meeting the above criteria were excluded.

### Study population and sample selection

The study was conducted among nurses and midwives employed in healthcare institutions operating at the tertiary level of the perinatal care referral system in the Silesian Voivodeship. This specifically defined professional group constitutes the actual study population in this research project.

For the purpose of estimating the required sample size, reference was initially made to the total number of registered nurses and midwives in the Silesian Voivodeship in 2023, which amounted to 44,950 individuals^19^. A conservative assumption of maximum uncertainty (*P* = 0.5) was applied, resulting in the largest required sample size. Assuming a 95% confidence level (Z = 1.96) and a maximum allowable margin of error of 5% (e = 0.05), the minimum sample size was calculated using the formula for a finite population:

$${\text{n}} = \frac{{{\text{N }} \times {\text{ Z}^{2}}{\text{ }} \times {\text{ P }} \times {\text{ }}\left( {{\text{1 }} - {\text{ P}}} \right)}}{{\left( {{\text{N }} - {\text{ 1}}} \right){\text{ }} \times {\text{ e}^{2}}{\text{ }} + {\text{ Z}^{2}}{\text{ }} \times {\text{ P }} \times {\text{ }}\left( {{\text{1 }} - {\text{ P}}} \right)}}$$

where:

- *n* – required sample size,
- *N* – population size (44,950),
- *Z* – value from the standard normal distribution corresponding to the confidence level (1.96),
- *P* – assumed proportion of the characteristic in the population (0.5),
- *e* – maximum allowable margin of error (0.05).

The result of this calculation indicated that a minimum of 381 respondents was needed. Ultimately, 307 correctly completed questionnaires were included in the analysis. Despite the final number being slightly lower than the initially estimated minimum, the sample was deemed representative of the target group due to the highly specific nature of the study population—namely, nurses and midwives working exclusively within tertiary-level perinatal care institutions.

## Ethical approval

According to the decision of the Bioethics Committee of the Medical University of Silesia in Katowice (decision no. PCN/CBN/0052/KB/33/23 dated 28 March 2023), the study was not classified as a medical experiment and therefore did not require formal ethical approval.

### Description of the research instrument

The original questionnaire consisted of two parts. The first part included questions on sociodemographic variables (age, sex, profession, place of employment, total work experience, and length of service at the current workplace). The second part concerned psychological and professional aspects, including: frequency of contact with patients with adverse prenatal diagnoses, presence during the death of newborns, frequency of care for children with lethal anomalies, types of emotions experienced, levels of subjective discomfort, and identification with specific personality traits.

Respondents could select multiple emotional responses (e.g. sadness, helplessness, compassion, calmness, anger, uncertainty, rage), and rate their intensity on a six-point Likert scale (from “none” to “very strong”). In the question on personality traits (e.g. empathy, psychological resilience, patience, engagement), respondents chose from a closed list of options.

### Data processing and statistical analysis

The data underwent a cleaning process, including elimination of incomplete or inconsistent responses, logic checks, and identification of missing values. Responses were then numerically coded, with multiple-choice questions converted into binary format (0 = not selected, 1 = selected) and entered into a Microsoft Excel spreadsheet.

Statistical analyses were performed using Microsoft Excel and Jamovi software (v2.4.8). The following procedures were applied:

1. Descriptive statistics – arithmetic means, standard deviations, medians, and ranges (min–max),
2. Frequency analysis – for categorical variables,
3. Shapiro–Wilk test – to assess normality of distribution,
4. Correlation analysis – Pearson’s r for normally distributed data and Spearman’s ρ for ordinal or non-normally distributed data,
5. Non-parametric tests – Mann–Whitney U test and Kruskal–Wallis test, depending on the number of compared groups.

All tests were conducted at a significance level of α = 0.05. Results with p-values < 0.05 were considered statistically significant. The findings were interpreted with consideration of the clinical and psychological context of healthcare work in emotionally demanding conditions^4^.

## Results

### Characteristics of the study group

A total of 307 individuals participated in the study, the vast majority of whom were female (99%). Participants were fairly evenly distributed across age categories, with roughly one-third aged 40 years or younger, one-third between 41 and 50 years, and one-third aged 51 years and older. The overall length of professional experience was varied, with a slightly higher proportion of participants having 27–31 years of employment or less than 10 years.

Tenure in the current ward also varied, with the largest group comprising those employed for up to 5 years (24.8%), followed by those with 12–17 years of tenure (21.8%). Regarding the workplace, most participants were employed in neonatal intensive care units (39.4%) or neonatology wards (27.7%). Smaller proportions worked in obstetric wards (16.3%), delivery wards (13.4%), or perinatal hospices (3.3%).

In terms of professional role, midwives predominated, accounting for over 60% of the sample, while nurses represented 37.1%. A small minority (1.3%) were qualified as both nurse and midwife. Detail present in Table 1.

**Table 1:** Sociodemographic characteristics of the study partipants (*n* = 307).

Variable	Category	Number (*n*)	Percentage (%)
Sex	Female	304	99.0%
	Male	3	1.0%
Age	≤ 40 years	105	34.2%
	41–50 years	104	33.9%
	≥ 51 years	98	31.9%
Total years of employment	≤ 10 years	65	21.2%
	11–19 years	65	21.2%
	20–26 years	59	19.2%
	27–31 years	72	23.5%
	≥ 32 years	46	15.0%
Tenure in current ward	≤ 5 years	76	24.8%
	6–11 years	52	17.0%
	12–17 years	67	21.8%
	18–26 years	56	18.2%
	≥ 27 years	56	18.2%
Workplace	Delivery ward	41	13.4%
	Perinatal hospice	10	3.3%
	Neonatal intensive care unit (NICU)	121	39.4%
	Neonatology ward	85	27.7%
	Obstetric ward	50	16.3%
Profession	Nurse	114	37.1%
	Midwife	189	61.6%
	Nurse and midwife	4	1.3%

## Type and intensity of emotions experienced by staff caring for newborns with lethal anomalies

The emotional experiences of staff caring for newborns with lethal anomalies vary in intensity, with compassion, sadness, and helplessness being the most frequently reported emotions.

### Very intense emotions

Compassion was the most frequently reported very intense emotion (28.1%), followed by sadness/despair (16.9%) and helplessness/powerlessness (19.2%). These findings suggest a significant amount of empathy and emotional pain, with many respondents feeling overwhelmed by the circumstances. In contrast, fear of death (8.1%) and anger (9.4%) were reported less intensely, indicating that while these emotions exist, they are not as prominent. Indifference was very rarely reported (0.7%), highlighting that emotional detachment is uncommon.

### Strong emotions

Compassion remained the dominant strong emotion (49.7%), with grief/pity (40.1%) and sadness (37.1%) following closely. These emotions reflect empathy and sorrow toward both others and personal circumstances. Fear of death (20.5%) and helplessness (31.9%) were also notable at this level, pointing to existential anxiety and a sense of vulnerability. Anger (8.1%) and indifference (5.5%) were less prevalent, suggesting that frustration and emotional withdrawal are not as widespread.

### Moderate emotions

At moderate intensity, calmness (37.1%) emerged as the most common emotion, followed by sadness (30.6%) and hope (19.5%). This suggests that many participants are able to maintain some emotional stability despite difficult situations. Indifference (14.0%) was less common at this level, indicating that emotional detachment was not the primary coping strategy.

### Mild emotions

Hope (31.3%) was the most prominent mild emotion, reflecting a degree of optimism despite the adversity faced. Relief (22.5%) and despair (19.9%) were also reported, though they did not dominate. Interestingly, fear of death was experienced more often at this level (28.0%) than at higher intensities, showing that existential concerns, while present, are often not overwhelming.

### Minimal emotions

At minimal intensity, indifference (15.0%) and hope (20.2%) were the most common emotions. These findings suggest that some participants may emotionally distance themselves or retain minimal optimism, but these emotions remain subdued. Minimal sadness (2.3%) and grief (5.2%) were rarely reported, indicating that these feelings, when experienced, tend to be more intense.

### Absence of emotions

**Fig. 1:**
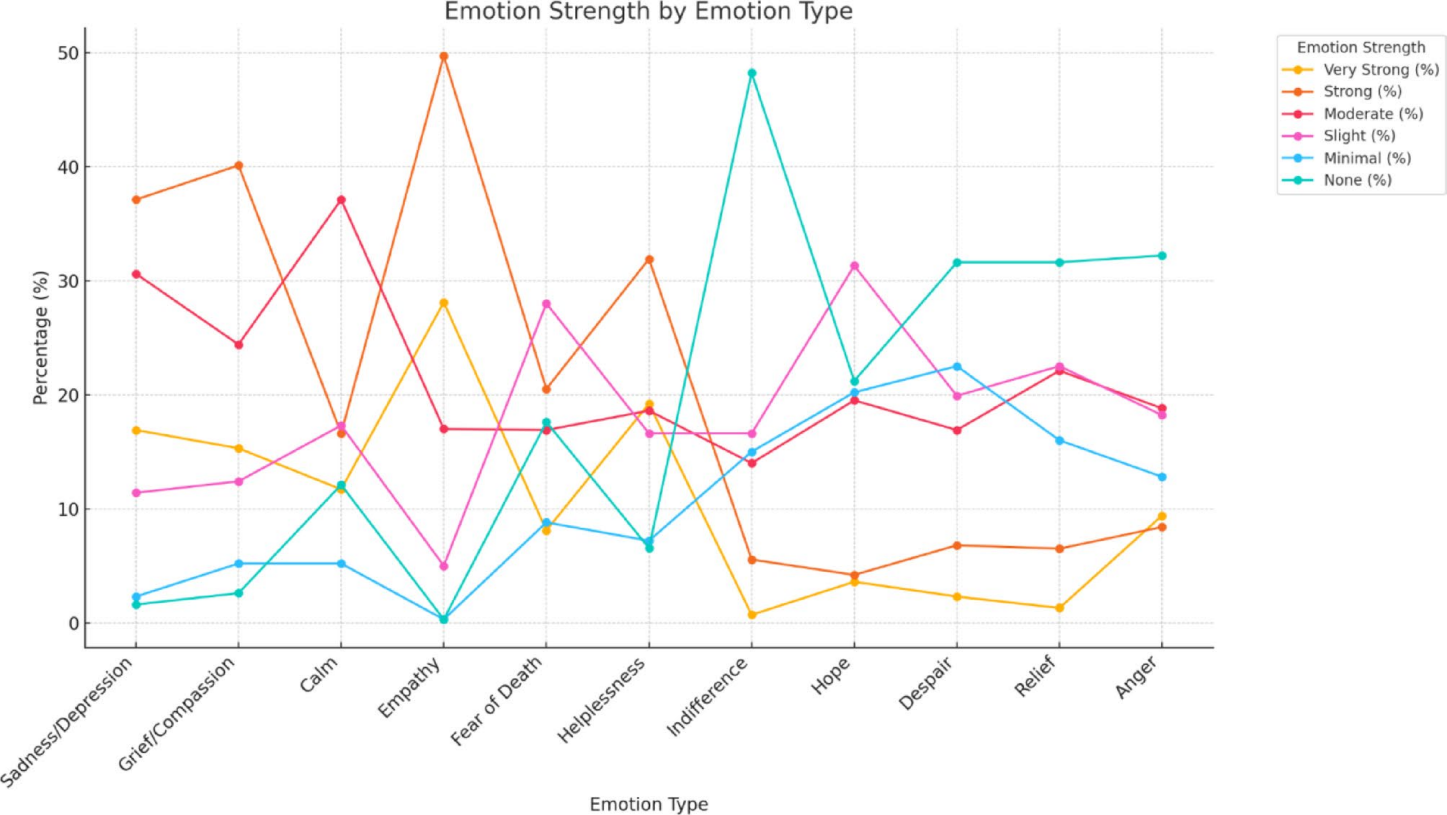
Emotion Strenght by Emotion Type.

A significant number of participants reported no experience of indifference (48.2%) or despair (31.6%), suggesting emotional engagement and hope despite challenges. Many also reported no lack of calmness (12.1%) or relief (31.6%), which may reflect persistent anxiety or an inability to alleviate emotional tension. Notably, the absence of anger (32.2%) and helplessness (6.5%) indicated that these emotions were less common in the respondents’ experiences. Figure 1.

This analysis illustrates the complex emotional landscape faced by staff, with compassion, sadness, and helplessness being dominant, while fear of death and anger play a lesser role.

### Emotional correlates of professional experience

The analysis of Spearman’s rank correlations revealed several noteworthy associations between emotional responses and years of professional experience. Although the majority of the correlations did not reach statistical significance, some patterns emerged that may have practical relevance due to the magnitude of the effect sizes.

Most notably, sadness was positively associated with both total years of professional experience (*r* = 0.20, *p* < 0.001) and years of experience in the current unit (*r* = 0.24, *p* < 0.001), both with effect sizes classified as weak but consistent and statistically robust. This may suggest a cumulative emotional impact associated with prolonged exposure to clinical stressors and end-of-life care, highlighting a potential vulnerability to emotional exhaustion or compassion fatigue over time.

Anger demonstrated a statistically significant negative correlation with total professional experience (*r* = − 0.15, *p* = 0.008) and a marginally significant correlation with experience in the current unit (*r* = − 0.11, *p* = 0.050). Although weak in magnitude, these results may reflect enhanced emotion regulation skills among more experienced staff.

Additionally, hope (*r* = − 0.12, *p* = 0.037) and indifference (*r* = − 0.12, *p* = 0.034) showed weak but significant negative associations with experience. These findings may suggest a gradual decline in positive outlook and emotional detachment as potential psychological adaptations to sustained stress, which could either support coping or contribute to depersonalisation.

Other emotional variables (e.g., helplessness, fear of death, calmness, relief, compassion, regret/pity, despair) were not significantly correlated with professional experience, and all effect sizes were negligible (*r* < 0.10), indicating minimal or no linear relationship. Nevertheless, the absence of statistical significance does not exclude the possibility of complex, non-linear effects or interactions with contextual factors not captured in the current analysis. Table 2.

**Table 2:** Correlations between emotional varables and work experience.

Emotion	*n*	Overall Work Experience				Work Experience in Current Unit			
		*r*	95% CI	Strength of correlation	*p*	*r*	95% CI	Strength of correlation	*p*
Helplessness	307	0.05	[−0.06, 0.16]	Negligible	0.402	−0.02	[−0.14, 0.09]	Negligible	0.673
Anger	307	**−0.15**	[−0.26, −0.04]	Weak	**0.008**	**−0.11**	[−0.22, 0.00]	Weak	**0.050**
Fear of death	307	0.08	[−0.03, 0.19]	Negligible	0.166	0.05	[−0.06, 0.16]	Negligible	0.375
Thought of inadequate patient care	307	−0.07	[−0.18, 0.04]	Negligible	0.198	−0.09	[−0.20, 0.02]	Negligible	0.109
Hope	307	−0.11	[−0.22, 0.00]	Weak	0.055	**−0.12**	[−0.23, −0.01]	Weak	**0.037**
Indifference	307	**−0.12**	[−0.23, −0.01]	Weak	**0.034**	−0.04	[−0.16, 0.07]	Negligible	0.443
Sadness	307	**0.20**	[0.09, 0.30]	Weak	**< 0.001**	**0.24**	[0.13, 0.34]	Weak	**< 0.001**
Despair	307	−0.01	[−0.12, 0.10]	Negligible	0.820	−0.04	[−0.15, 0.07]	Negligible	0.458
Calmness	307	0.04	[−0.08, 0.15]	Negligible	0.520	0.09	[−0.03, 0.20]	Negligible	0.128
Relief	307	0.06	[−0.05, 0.17]	Negligible	0.293	0.09	[−0.02, 0.20]	Negligible	0.111
Compassion	307	0.03	[−0.09, 0.14]	Negligible	0.643	0.02	[−0.09, 0.13]	Negligible	0.699
Regret/Pity	307	0.02	[−0.09, 0.13]	Negligible	0.719	0.09	[−0.02, 0.20]	Negligible	0.117

*n* – number of participants included in the analysis (sample size), *r* – Spearman’s rank correlation coefficient between the emotion and the respondent’s total years of professional experience, 95% CI – 95% Confidence Interval for the correlation coefficient. Provides a range within which the true correlation coefficient is likely to fall with 95% certainty, *p* – p-value associated with the correlation, *p* < 0.05 denotes a statistically significant correlation. Strength of correlation: 0.00- 0.10 – Negligible, 0.10–0.29 – Weak, 0.30–0.49 – Moderate, 0.50–1.00 – Strong.

### Emotional correlates of clinical exposure

Several statistically and practically relevant correlations were observed between emotional responses and variables related to clinical exposure. The most prominent finding was the strong, positive correlation between sadness and the level of discomfort associated with caring for neonates with a lethal condition (ρ = 0.505, *p* < 0.001). This was the only correlation classified as strong in the entire dataset and underscores the emotional toll of perinatal palliative care.

Moderate positive correlations were also found between discomfort and both regret/pity (ρ = 0.391, *p* < 0.001) and fear of death (ρ = 0.312, *p* < 0.001), indicating that these emotional responses may significantly contribute to clinicians’ psychological burden in end-of-life contexts.

Several additional emotional states showed weak but statistically significant positive correlations with discomfort:

- Despair (ρ = 0.261, *p* < 0.001),
- Anger (ρ = 0.154, *p* = 0.007),
- Hope (ρ = 0.128, *p* = 0.025),
- Compassion (ρ = 0.225, *p* < 0.001),
- Helplessness (ρ = 0.198, *p* < 0.001).

These findings suggest that the experience of discomfort during palliative care is emotionally multifaceted and shaped by both negative and prosocial responses.

Conversely, calmness was negatively correlated with discomfort (ρ = − 0.180, *p* = 0.002), potentially reflecting a protective effect of emotional stability or mindfulness.

Other significant associations emerged between frequency-based exposure variables and emotions such as anger, despair, relief, and fear of death, all exhibiting weak but consistent correlations (ρ = 0.13–0.26, *p* < 0.05). These results support the notion that repeated exposure to ethically complex or emotionally charged scenarios may heighten affective responses, albeit with considerable interindividual variability. Details present in Table 3.

**Table 3:** Spearman’s Rho correlations between emotions and selected clinical exposure variables.

Emotion	Frequency of Admitting Patients with a Prenatally Diagnosed Lethal Condition	Frequency of Contact with Neonatal Death and Dying	Frequency of Performing Nursing Care Procedures for Neonates with Lethal Conditions	Level of Discomfort Associated with Care for Neonates with Lethal Conditions
**Sadness**	ρ = 0.020 (Negligible) *p* = 0.728	ρ = 0.035 (Negligible) *p* = 0.538	ρ = −0.057 (Negligible) *p* = 0.321	**ρ = 0.505** (Strong) *p* **< 0.001**
**Regret/Pity**	ρ = 0.026 (Negligible) *p* = 0.654	ρ = 0.058 (Negligible) *p* = 0.315	ρ = −0.067 (Negligible) *p* = 0.245	**ρ = 0.391** (Moderate) *p* **< 0.001**
**Calmness**	ρ = 0.063 (Negligible) *p* = 0.272	ρ = 0.052 (Negligible) *p* = 0.363	ρ = 0.105 (Weak) *p* = 0.065	**ρ = −0.180** (Weak) *p* **= 0.002**
**Compassion**	**ρ = 0.147** (Weak) *p* **= 0.010**	ρ = 0.070 (Negligible) *p* = 0.225	ρ = 0.108 (Weak) *p* = 0.060	**ρ = 0.225** (Weak) *p* **< 0.001**
**Fear of Death**	**ρ = 0.148** (Weak) *p* **= 0.009**	**ρ = 0.135** (Weak) *p* **= 0.018**	ρ = 0.097 (Negligible) *p* = 0.090	**ρ = 0.312** (Moderate) *p* **< 0.001**
**Helplessness**	ρ = −0.009 (Negligible) *p* = 0.876	ρ = 0.044 (Negligible) *p* = 0.446	ρ = 0.086 (Negligible) *p* = 0.133	**ρ = 0.198** (Weak) *p* **< 0.001**
**Indifference**	ρ = −0.045 (Negligible) *p* = 0.428	ρ = 0.022 (Negligible) *p* = 0.698	**ρ = −0.124** (Weak) *p* **= 0.029**	ρ = −0.057 (Negligible) *p* = 0.323
**Hope**	ρ = 0.051 (Negligible) *p* = 0.371	ρ = 0.062 (Negligible) *p* = 0.277	**ρ = −0.116** (Weak) *p* **= 0.042**	**ρ = 0.128** (Weak) *p* **= 0.025**
**Despair**	**ρ = 0.150** (Weak) *p* **= 0.008**	**ρ = 0.174** (Weak) *p* **= 0.002**	ρ = 0.038 (Negligible) *p* = 0.505	**ρ = 0.261** (Weak) *p* **< 0.001**
**Relief**	**ρ = 0.141** (Weak) *p* **= 0.013**	**ρ = 0.159** (Weak) *p* **= 0.005**	ρ = 0.058 (Negligible) *p* = 0.315	ρ = −0.007 (Negligible) *p* = 0.903
**Anger**	**ρ = 0.236** (Weak) *p* **< 0.001**	**ρ = 0.257** (Weak) *p* **< 0.001**	ρ = 0.059 (Negligible) *p* = 0.304	**ρ = 0.154** (Weak) *p* **= 0.007**
**Thought of Inadequate Patient Care**	ρ = 0.075 (Negligible) *p* = 0.190	**ρ = 0.134** (Weak) *p* **= 0.018**	ρ = −0.005 (Negligible) *p* = 0.933	ρ = 0.062 (Negligible) *p* = 0.281

ρ = Spearman’s rank correlation coefficient, p = Significance value, Statistically significant values (*p* < 0.05) are bolded. Strength of correlation: 0.00- 0.10 – Negligible, 0.10–0.29 – Weak, 0.30–0.49 – Moderate, 0.50–1.00 – Strong.

## Results of descriptive statistics for personal characteristics

The analysis of descriptive statistics for the 307 participants revealed generally high ratings across the assessed traits, with most variables showing means near the upper end of the scale (1–3). Medians for all traits were 2.00, indicating a central tendency toward moderate-to-high endorsement of each characteristic. Distributions were symmetrical and platykurtic, suggesting flattened curves compared to the normal distribution.

### Central tendency and dispersion

The highest-rated traits were empathic and compassionate (M = 2.06, SD = 0.79), caring (M = 2.05, SD = 0.80), kind (M = 2.05, SD = 0.81), honest (M = 2.02, SD = 0.82), and sensitive (M = 2.02, SD = 0.78), reflecting a strong orientation toward relational and ethical dimensions of care. Traits like psychologically strong (M = 1.95, SD = 0.75) and communicative (M = 1.94, SD = 0.73) also scored positively, indicating competence in interpersonal and intrapersonal skills.

Lower scores were observed for assertive (M = 1.66, SD = 0.73) and meticulous (M = 1.75, SD = 0.75), though their medians still indicated moderate agreement. The lowest mean was for reliable (M = 1.34, SD = 0.56), with a median of 1.00, suggesting this trait was less strongly endorsed or interpreted narrowly by respondents.

### Distributional characteristics

Most traits showed negative kurtosis, indicating distributions flatter than the normal curve, consistent with ceiling effects in traits often endorsed at high levels. Skewness values ranged from − 0.11 (compassion) to + 1.41 (reliable). Reliable showed strong right-skewness, suggesting most participants rated themselves low on this item, while a smaller group rated themselves much higher. Assertiveness showed mild right-skewness (skew = 0.62), indicating less consistent endorsement. Table 4.

### Summary

The data highlight that respondents strongly identify with prosocial traits such as compassion and kindness, while traits related to self-confidence and dependability, like assertiveness and reliability, were rated lower and showed more variability. These findings suggest areas for professional development, particularly in communication assertiveness and reliability in task execution.

**Table 4:** Descriptive statistics for personal characteristics.

Variable	*N*	M	SD	Median	Min.	Max.	Skewness	Kurtosis
psychologically strong	307	1.95	0.750	2.00	1.00	3.00	0.07	−1.22
selfless	307	1.92	0.796	2.00	1.00	3.00	0.14	−1.42
caring	307	2.05	0.803	2.00	1.00	3.00	−0.08	−1.45
open-minded	307	1.89	0.730	2.00	1.00	3.00	0.18	−1.12
empathic and compassionate	307	2.06	0.792	2.00	1.00	3.00	−0.11	−1.40
kind	307	2.05	0.805	2.00	1.00	3.00	−0.09	−1.46
sensitive	307	2.02	0.782	2.00	1.00	3.00	−0.03	−1.37
patient	307	1.92	0.776	2.00	1.00	3.00	0.13	−1.33
honest	307	2.02	0.820	2.00	1.00	3.00	−0.04	−1.52
assertive	307	1.66	0.734	2.00	1.00	3.00	0.62	−0.93
reliable	307	1.34	0.563	1.00	1.00	3.00	1.41	0.99
communicative	307	1.94	0.732	2.00	1.00	3.00	0.09	−1.14
meticulous and detail-oriented	307	1.75	0.749	2.00	1.00	3.00	0.4	−1.11

**N** – sample size, **M** – mean, **SD** – standard deviation, **Min./Max.** – minimum and maximum observed values, **Skewness** – symmetry of distribution (positive = right-skewed), **Kurtosis** – peakedness of distribution (negative = flatter than normal distribution).

## Personality traits and professional experience

Among the personality traits examined, only **empathic/compassionate** (*r* = 0.12, *p* = 0.034) and **open-minded** (*r* = 0.11, *p* = 0.048) dispositions were significantly, albeit weakly, associated with total professional experience. These findings may suggest that traits facilitating interpersonal understanding and cognitive flexibility contribute to professional longevity in emotionally demanding healthcare settings. Details present in Table 5.

No significant associations were observed between personality traits and years of experience in the current unit, indicating that individual differences may influence broader career trajectories more than stability within a particular workplace.

**Table 5:** Pearson’s r correlations between personal characteristics and professional Experience.

Personal Characteristic	Total Experience				Experience in Current Ward			
	*r*	95% CI (Lower; Upper)	Strength of correlation	*p*-value	*r*	95% CI (Lower; Upper)	Strength of correlation	*p*-value
psychologically strong	−0.10	[−0.21; 0.01]	Weak	0.089	0.05	[−0.06; 0.16]	Negligible	0.388
reliable	0.00	[−0.12; 0.11]	Negligible	0.934	−0.01	[−0.12; 0.10]	Negligible	0.892
caring	−0.09	[−0.20; 0.02]	Negligible	0.118	0.02	[−0.09; 0.13]	Negligible	0.734
honest	−0.09	[−0.20; 0.02]	Negligible	0.103	−0.09	[−0.20; 0.02]	Negligible	0.103
sensitive	0.05	[−0.06; 0.16]	Negligible	0.393	0.05	[−0.06; 0.16]	Negligible	0.393
kind	0.05	[−0.06; 0.16]	Negligible	0.403	0.05	[−0.06; 0.16]	Negligible	0.403
empathic/compassionate	**0.12**	[0.01; 0.23]	Weak	0.034*	0.03	[−0.08; 0.15]	Negligible	0.557
open-minded	**0.11**	[0.00; 0.22]	Weak	0.048*	0.02	[−0.09; 0.13]	Negligible	0.698
meticulous/detail-oriented	0.01	[−0.10; 0.12]	Negligible	0.888	−0.04	[−0.15; 0.08]	Negligible	0.532
communicative	0.03	[−0.08; 0.14]	Negligible	0.602	−0.09	[−0.20; 0.03]	Negligible	0.134
patient	0.10	[−0.01; 0.21]	Weak	0.071	0.03	[−0.08; 0.14]	Negligible	0.591
selfless	0.08	[−0.03; 0.19]	Negligible	0.162	0.04	[−0.07; 0.15]	Negligible	0.508
assertive	−0.01	[−0.12; 0.10]	Negligible	0.849	−0.07	[−0.19; 0.04]	Negligible	0.190

r – Pearson correlation coefficient, 95% CI – 95% Confidence Interval (Lower, Upper bounds), p – p-value (statistical significance), Sign. – significance markers: *p* < 0.05, ** *p* < 0.01, *** *p* < 0.001. Strength of correlation: 0.00- 0.10 – Negligible, 0.10–0.29 – Weak, 0.30–0.49 – Moderate, 0.50–1.00 – Strong.

## Personality traits and clinical exposure

Although most correlations between personality traits and clinical exposure variables were weak in magnitude, several statistically significant associations were identified:

- Open-mindedness was positively associated with the frequency of performing nursing care procedures (*r* = 0.148, *p* = 0.009), suggesting a greater willingness to engage with complex clinical responsibilities.
- Kindness correlated positively with both procedural frequency (*r* = 0.122, *p* = 0.033) and perceived discomfort (*r* = 0.126, *p* = 0.027), potentially indicating that affectively sensitive individuals may be more actively involved in care but also more emotionally impacted by it.
- Meticulousness showed a positive association with the frequency of contact with neonatal death (*r* = 0.114, *p* = 0.046), which may reflect heightened responsibility or conscientiousness in challenging clinical contexts.
- Communicativeness was positively correlated with the frequency of procedures (*r* = 0.123, *p* = 0.031), consistent with the role of interpersonal engagement in team-based care delivery.

Other traits, including selflessness, assertiveness, sensitivity, and psychological strength, did not show significant relationships with exposure variables, and their effect sizes were negligible. However, these null results may reflect contextual moderation or require larger sample sizes to detect subtle effects. Table 6.

**Table 6:** Spearman’s Rho correlations between declared personal traits and aspects of clinical Exposure.

Personal Trait	Frequency of admitting patients with a prenatally diagnosed lethal condition	Frequency of contact with neonatal death and dying	Frequency of performing nursing procedures related to the care of newborns with a lethal condition	Level of perceived discomfort associated with caring for a newborn with a lethal condition
**Psychologically strong**	*r* = − 0.096 (Negligible) (*p* = 0.092)	*r* = − 0.036 (Negligible) (*p* = 0.532)	*r* = 0.096 (Negligible) (*p* = 0.095)	*r* = 0.035 (Negligible) (*p* = 0.543)
**Selfless**	*r* = − 0.082 (Negligible) (*p* = 0.151)	*r* = − 0.024 (Negligible) (*p* = 0.679)	*r* = 0.032 (Negligible) (*p* = 0.580)	*r* = 0.070 (Negligible) (*p* = 0.221)
**Caring**	*r* = − 0.045 (Negligible) (*p* = 0.428)	*r* = − 0.092 (Negligible) (*p* = 0.108)	*r* = − 0.006 (Negligible) (*p* = 0.915)	*r* = 0.166 (Weak) **(*p*** = 0.004)**
**Open-minded**	*r* = − 0.074 (Negligible) (*p* = 0.197)	*r* = 0.002 (Negligible) (*p* = 0.979)	*r* = 0.148 (Weak) **(*p*** = 0.009)**	*r* = 0.087 (Negligible) (*p* = 0.129)
**Empathic/compassionate**	*r* = 0.056 (Negligible) (*p* = 0.325)	*r* = − 0.057 (Negligible) (*p* = 0.321)	*r* = 0.030 (Negligible) (*p* = 0.599)	*r* = 0.099 (Negligible) (*p* = 0.083)
**Kind**	*r* = 0.006 (Negligible) (*p* = 0.916)	*r* = 0.000 (Negligible) (*p* = 0.994)	*r* = 0.122 (Negligible) **(*p*** = 0.033)*	*r* = 0.126 (Weak) **(*p*** = 0.027)*
**Sensitive**	*r* = − 0.052 (Negligible) (*p* = 0.363)	*r* = − 0.027 (Negligible) (*p* = 0.636)	*r* = 0.004 (Negligible) (*p* = 0.947)	*r* = 0.093 (Negligible) (*p* = 0.102)
**Patient**	*r* = 0.074 (Negligible) (*p* = 0.193)	*r* = − 0.027 (Negligible) (*p* = 0.637)	*r* = 0.088 (Negligible) (*p* = 0.123)	*r* = 0.019 (Negligible) (*p* = 0.747)
**Honest**	*r* = 0.016 (Negligible) (*p* = 0.779)	*r* = 0.066 (Negligible) (*p* = 0.252)	*r* = 0.033 (Negligible) (*p* = 0.566)	*r* = 0.088 (Negligible) (*p* = 0.125)
**Assertive**	*r* = − 0.057 (Negligible) (*p* = 0.316)	*r* = 0.012 (Negligible) (*p* = 0.838)	*r* = 0.099 (Negligible) (*p* = 0.083)	*r* = 0.029 (Negligible) (*p* = 0.614)
**Reliable**	*r* = − 0.121 (Weak) **(*p*** = 0.034)*	*r* = 0.092 (Negligible) (*p* = 0.107)	*r* = − 0.002 (Negligible) (*p* = 0.968)	*r* = 0.022 (Negligible) (*p* = 0.700)
**Communicative**	*r* = 0.052 (Negligible) (*p* = 0.362)	*r* = 0.050 (Negligible) (*p* = 0.379)	*r* = 0.123 (Weak) **(*p*** = 0.031)*	*r* = 0.014 (Negligible) (*p* = 0.801)
**Meticulous/detail-oriented**	*r* = 0.047 (Negligible) (*p* = 0.413)	*r* = 0.114 (Weak) **(*p*** = 0.046)*	*r* = 0.042 (Negligible) (*p* = 0.465)	*r* = − 0.040 (Negligible) (*p* = 0.483)

**r** – Spearman’s rank correlation coefficient, **p** – p-value (significance level), *p* < 0.05 — statistically significant, ** *p* < 0.01 — highly statistically significant. Strength of correlation: 0.00- 0.10 – **Negligible**, 0.10–0.29 – **Weak**, 0.30–0.49 – **Moderate**, 0.50–1.00 – **Strong**.

## Discussion

A review of the literature has revealed a significant research gap: although numerous studies have explored the emotional experiences of nurses and midwives, there is a lack of in-depth analyses concerning the impact of caring for newborns with life-limiting conditions on the emotional wellbeing of medical personnel. Existing research tends to focus on general emotional responses to challenging clinical situations, without specifically addressing perinatology or neonatal palliative care^20,21^.

This study focuses on analysing the type and intensity of emotions experienced by staff during the care of neonates with lethal anomalies, as well as identifying the personality traits with which nurses and midwives most closely identify. The study also examined the relationships between these variables and factors such as the frequency of contact with patients receiving an adverse prenatal diagnosis, the number of cases involving neonatal death, and the level of reported psychological discomfort.

The literature review confirmed that neither the structure of emotional experiences nor the personality profiles associated with caring for newborns with limited life expectancy have been comprehensively examined in empirical research to date. Therefore, this discussion also considers findings from related studies on emotions and personality traits in palliative and end-of-life care in other medical contexts^22,23^.

This study represents an attempt to holistically assess these aspects and aims to contribute to the existing body of knowledge regarding the psychological dimensions of healthcare work under high emotional strain in perinatal care.

The analysis suggests that the emotional experiences of medical staff working with neonates with life-limiting conditions are multidimensional, and their intensity and direction depend on both individual characteristics and environmental factors. Literature indicates that emotions such as sadness, compassion, helplessness, and grief are among the most frequently experienced in the care of terminally ill patients. Research by Kostka et al.^24^ and André et al.^25^ shows that such emotions dominate in palliative care and can significantly affect staff mental wellbeing. Similar findings were reported by Khalaf et al.^26^ and Guo et al.^27^, particularly in the context of oncology nursing, where emotions such as sadness and fear of death are particularly pronounced.

The study found that the predominant emotions were sadness and despair, experienced by respondents at strong or very strong levels. Emotions such as grief and helplessness were reported at moderate levels, while fear of death, hope, despair, or anger were infrequent, and indifference was virtually absent. These results align with the literature; however, it is worth emphasising that emotions in the context of neonatal care may differ from those experienced in adult care. Newborns are unable to communicate verbally, which adds to the challenge and increases the psychological burden, as the nurse or midwife must not only interpret physical symptoms and behaviours but also act as an emotional intermediary between the infant and the family. This unique position fosters a special bond and empathy, which can intensify emotional experiences^25,27,28^.

Personality traits of healthcare personnel were found to play a significant role in shaping emotional experiences. The most commonly identified traits among respondents were empathy, compassion, kindness, sensitivity, and honesty. While international studies often highlight traits such as agreeableness, conscientiousness, psychological resilience, and independence^29–38^, the findings of this study indicate that, within the Polish cultural context and the specific demands of perinatal care, empathetic traits are more prominent. Complementary findings by Chen et al.^32^ and Lalonde et al.^36^ suggest that nurses’ professional experience is associated with increased empathy and a reduced fear of death. These results point to the possibility of positive adaptation over the course of one’s career, while also highlighting the risk of excessive emotional involvement, which—over time—can deplete psychological resources and lead to burnout^39^.

One of the more concerning findings of this study was a decline in hope and an increasing sense of sadness among more experienced staff. While experience can foster greater psychological resilience and coping skills, prolonged exposure to extreme situations may lead to chronic emotional overload. This is also supported by findings from Vallone et al.^40^, which show that nurses with many years of professional experience are more prone to depression and pessimism, despite maintaining a high level of professional engagement and responsibility^41^.

The analysis of the relationship between professional experience and emotional reactions revealed that negative emotions such as anger, anxiety, and despair occurred more frequently in individuals with regular contact with neonates with lethal anomalies. At the same time, a higher number of neonatal deaths appeared to reduce the level of fear of death, in line with findings by Üstüküş et al.^42^ and Fitch^43^. This points to the phenomenon of emotional habituation, an adaptive mechanism for coping with traumatic realities. However, excessive habituation may lead to reduced empathy and increased indifference, which can negatively affect the quality of care^23^.

A particularly noteworthy aspect of the study was the coexistence of both positive and negative emotions, indicating the emotional ambivalence inherent in caregiving work. Nurses and midwives, despite being acutely aware of the inevitability of neonatal death, also reported feelings of fulfilment, satisfaction, and love. Providing dignified conditions for dying, being present with the infant during its final moments, and offering support to the family all evoked positive emotions that may serve as a counterbalance to suffering and frustration^44,45^. Within the framework of post-traumatic growth (PTG) theory, such experiences may be seen as contributing to personal and professional growth in response to confronting death, a notion also supported by Barnett et al.^46^.

Attention should also be drawn to the phenomenon of moral distress, which often accompanies the care of neonates with life-limiting conditions. Medical personnel may find themselves required to carry out procedures or clinical decisions that conflict with their personal values or moral convictions. This creates intense internal tension which, if unsupported, can lead to chronic burnout and a decision to leave the field. The literature increasingly calls for systemic support for healthcare professionals, including regular supervision, communication training, and psychological interventions^20,21,39^.

Finally, it is important to note that, despite the difficult working conditions, a significant proportion of participants reported an absence of emotions such as indifference or despair. This indicates a high level of emotional engagement and psychological resilience, which may stem from both personality traits and effective coping mechanisms. At the same time, the low number of respondents reporting feelings of calm or relief may suggest difficulties in emotional release, which, over time, could result in stress accumulation and decreased professional effectiveness^47,48^.

In conclusion, caring for neonates with life-limiting anomalies constitutes an exceptionally demanding and emotionally burdensome area of work for nurses and midwives. The emotions experienced in this context are complex, ambivalent, and closely tied to personality traits and professional experience. Prolonged exposure to extreme situations may lead to both positive professional development and emotional exhaustion, particularly in the absence of institutional support. The findings of this study highlight the urgent need for strategies to safeguard the mental health of medical staff, including psychological support, training, and ethical reflection. More broadly, this study addresses a significant gap in the literature by offering in-depth insights into the psychological dimensions of work in perinatal palliative care, while also suggesting directions for further research on the emotional wellbeing of healthcare professionals working under conditions of high emotional strain.

## Strengths of the study

This study presents several important strengths that enhance its scientific and practical value. It addresses a socially relevant issue: the emotional well-being of healthcare professionals providing care to neonates with life-limiting conditions. In the context of increasing awareness of occupational stress in medical settings, exploring emotions such as sadness, despair, empathy, and emotional fatigue is both timely and necessary. Rather than focusing solely on burnout, the authors adopt a broader perspective, suggesting that increased empathy and relational engagement may reflect professional growth rather than emotional desensitisation.

A key strength lies in the study’s comprehensive approach. It considers both individual characteristics and clinical exposure, allowing for the identification of emotional patterns. For instance, the negative correlation between empathy and the frequency of contact with dying patients may indicate the use of emotional distancing as a coping strategy. This raises important questions about staff well-being and the continuity of compassionate care.

The use of an original questionnaire, specifically tailored to the neonatal palliative care context, enabled the detection of emotional nuances that standardised tools might overlook. Although the tool has not been formally validated, its design reflects the realities of clinical work and supports practical relevance. The relatively large sample (*n* = 307) and diversity in institutional backgrounds and professional experience enhance the credibility of the findings.|.

Crucially, the study offers actionable conclusions. The authors recommend implementing structured emotional support – such as grief training, psychological assistance for long-serving staff, and rest zones in clinical settings. These proposals are supported by the observed decline in perceived mental resilience with years of service. Although not all emotional responses reached statistical significance, the identification of positive emotions such as hope or calm highlights their potential as psychological buffers.

### Limitations of the study

The study’s cross-sectional design limits causal interpretation. It cannot determine whether emotional strain results from exposure or a lack of institutional support. The original, non-validated questionnaire may impact the reliability and comparability of findings. Additionally, the sample was drawn from one region, limiting generalisability. As with most self-report research, the potential for socially desirable responses must also be acknowledged. Nonetheless, the study provides a valuable foundation for further research and practical improvement in staff support systems.

### Implications for practice and future research

In Poland, perinatal care faces significant systemic challenges that impact the mental health of healthcare professionals, particularly nurses and midwives. Most notably, access to psychological support for staff remains limited. Funding issues and a shortage of qualified specialists mean that the available assistance is often inadequate—especially in the fields of perinatology and neonatology, where emotional stress is particularly intense. The low availability of psychological support represents a critical gap that should be addressed in future research on support systems for healthcare workers.

There is also a lack of a formal mentoring system to assist younger staff—especially in perinatal wards—in their professional adaptation. Most early-career nurses and midwives express a need for support during their initial years of practice, yet structured mentoring or coaching programmes are largely absent. There is an urgent need to develop and implement such programmes to reduce the risk of burnout and to support staff in managing the emotional challenges associated with caring for seriously ill patients.

Another important issue is the absence of psychological screening for candidates entering the field of perinatology, where stress levels are particularly high. The current recruitment system does not assess psychological readiness, which may contribute to professional burnout. Approximately 35% of nurses struggle with this issue, negatively affecting the quality of care^49^. Introducing psychological assessments at the qualification stage could help improve staff wellbeing and reduce the risk of burnout.

In addressing the research questions related to these challenges, it is worth noting the limited access to psychological support in Poland. Only 17% of staff participate in group psychological consultations, with the majority relying on online resources^50^. Legislative changes are necessary to ensure that access to psychologists becomes a standard in healthcare institutions. Regarding mentoring, the pilot programme *“System of Incentives”*, launched in 2025, aims to support staff, but further comparative research is needed to evaluate the effectiveness of such initiatives^51,52^. Similarly, the introduction of psychological assessments during recruitment could help prevent mental health issues and improve the quality of care in high-stress environments such as perinatology.

The findings of this study highlight the need to evaluate the effectiveness of psychological support and mentoring programmes. Longitudinal studies should be undertaken to assess how such programmes influence perinatal staff by comparing supported groups with those not receiving support. It will also be important to understand the role of positive emotions—such as hope, calmness, or relief—in mitigating the emotional effects of working in healthcare. At the same time, further research should explore how healthcare institutions can support the mental health of staff by creating restorative spaces, implementing staff rotation, and ensuring easy access to psychological assistance.

In conclusion, systemic changes are urgently needed within the field of perinatal care to provide adequate psychological support for medical personnel, particularly in perinatology. Developing mentoring programmes, introducing psychological screening during recruitment, and improving access to mental health services are essential steps towards enhancing the quality of care and reducing the emotional burden on staff.

## Conclusions

The findings highlight several key relationships regarding the emotional burden experienced by healthcare workers. Firstly, extended professional experience does not eliminate emotional strain but may contribute to emotional stabilisation, thereby reducing the intensity of negative emotions such as anger and indifference. However, prolonged tenure in a single department is associated with an increase in feelings of sadness and emotional burnout, indicating the importance of professional diversity in providing psychological support for employees.

Subjective discomfort plays a critical role in emotional experiences, as greater emotional burden is more closely related to the perceived difficulty of caring for infants with lethal congenital anomalies, rather than the medical procedures themselves. This suggests that subjective perception, rather than solely objective challenges, largely shapes emotional responses in such situations.

One concerning finding is the low level of positive emotions among healthcare workers, such as hope, relief, and peace. These positive emotions occur infrequently or with reduced intensity, suggesting that their protective function is limited in the face of significant stress. This could indicate difficulties in emotional recovery in high-stress professions.

Furthermore, personality traits have a significant impact on emotional burden. Healthcare workers with more pronounced prosocial traits, such as empathy and sensitivity, tend to engage more deeply on an emotional level, which results in higher levels of stress and emotional burden. This suggests that while empathy is a crucial characteristic in caregiving professions, it may also increase vulnerability to burnout.

## Data Availability

The data generated during and analysed during the current study are available from the corresponding author on reasonable request.
